# First-In-Human Results on the Biodistribution, Pharmacokinetics, and Dosimetry of [^177^Lu]Lu-DOTA.SA.FAPi and [^177^Lu]Lu-DOTAGA.(SA.FAPi)_2_

**DOI:** 10.3390/ph14121212

**Published:** 2021-11-24

**Authors:** Sanjana Ballal, Madhav Prasad Yadav, Euy Sung Moon, Vasko S Kramer, Frank Roesch, Samta Kumari, Chandrasekhar Bal

**Affiliations:** 1 Department of Nuclear Medicine, AIIMS, Ansari Nagar, New Delhi 110029, India; mail.sanjanaballal87@gmail.com (S.B.); madhav_yadav2000@yahoo.com (M.P.Y.); kumarisamta97@gmail.com (S.K.); 2 Department of Chemistry, Johannes Gutenberg University, 55131 Mainz, Germany; emoon@students.uni-mainz.de (E.S.M.); froesch@uni-mainz.de (F.R.); 3 PositronPharma SA, Santiago 7500921, Chile; vkramer@positronpharma.cl

**Keywords:** [^68^Ga]Ga-DOTA.SA.FAPi PET/CT, [^177^Lu]Lu-DOTA.SA.FAPi, [^177^Lu]Lu-DOTAGA.(SA.FAPi)_2_, biodistribution, dosimetry, pharmacokinetics, absorbed dose estimates, effective half-life

## Abstract

Recently, great interest has been gained regarding fibroblast activation protein (FAP) as an excellent target for theranostics. Several FAP inhibitor molecules such as [^68^Ga]Ga-labelled FAPI-02, 04, 46, and DOTA.SA.FAPi have been introduced and are highly promising molecular targets from the imaging point of view. FAP inhibitors introduced via bifunctional DOTA and DOTAGA chelators offer the possibility to complex Lutetium-177 due to an additional coordination site, and are suitable for theranostic applications owing to the increased tumor accumulation and prolonged tumor retention time. However, for therapeutic applications, very little has been accomplished, mainly due to residence times of the compounds. In an attempt to develop a promising therapeutic radiopharmaceutical, the present study aimed to evaluate and compare the biodistribution, pharmacokinetics, and dosimetry of [^177^Lu]Lu-DOTA.SA.FAPi, and [^177^Lu]Lu-DOTAGA.(SA.FAPi)_2_ in patients with various cancers. The FAPi agents, [^177^Lu]Lu-DOTA.SA.FAPi and [^177^Lu]Lu-DOTAGA.(SA.FAPi)_2_, were administered in two different groups of patients. Three patients (mean age—50 years) were treated with a median cumulative activity of 2.96 GBq (IQR: 2.2–3 GBq) [^177^Lu]Lu-DOTA.SA.FAPi and seven (mean age—51 years) were treated with 1.48 GBq (IQR: 0.6–1.5) of [^177^Lu]Lu-DOTAGA.(SA.FAPi)_2_. Patients in both the groups underwent serial imaging whole-body planar and SPECT/CT scans that were acquired between 1 h and 168 h post-injection (p.i.). The residence time and absorbed dose estimate in the source organs and tumor were calculated using OLINDA/EXM 2.2 software. Time versus activity graphs were plotted to determine the effective half-life (Te) in the whole body and lesions for both the radiotracers. Physiological uptake of [^177^Lu]Lu-DOTA.SA.FAPi was observed in the kidneys, colon, pancreas, liver, gall bladder, oral mucosa, lacrimal glands, and urinary bladder contents. Physiological biodistribution of [^177^Lu]Lu-DOTAGA.(SA.FAPi)_2_ involved liver, gall bladder, colon, pancreas, kidneys, and urinary bladder contents, lacrimal glands, oral mucosa, and salivary glands. In the [^177^Lu]Lu-DOTA.SA.FAPi group, the highest absorbed doses were noted in the kidneys (0.618 ± 0.015 Gy/GBq), followed by the colon (right colon: 0.472 Gy/GBq and left colon: 0.430 Gy/GBq). In the [^177^Lu]Lu-DOTAGA.(SA.FAPi)_2_ group, the colon received the highest absorbed dose (right colon: 1.160 Gy/GBq and left colon: 2.870 Gy/GBq), and demonstrated a significantly higher mean absorbed dose than [^177^Lu]Lu-DOTA.SA.FAPi (*p* < 0.011). [^177^Lu]Lu-DOTAGA.(SA.FAPi)_2_ had significantly longer median whole-body Te compared to that of [^177^Lu]Lu-DOTA.SA.FAPi [46.2 h (IQR: 38.5–70.1) vs. 23.1 h (IQR: 17.8–31.5); *p*-0.0167]. The Te of tumor lesions was significantly higher for [^177^Lu]Lu-DOTAGA.(SA.FAPi)_2_ compared to [^177^Lu]Lu-DOTA.SA.FAPi [86.6 h (IQR: 34.3–94.6) vs. 14 h (IQR: 12.8–15.5); *p*-0.0004]. The median absorbed doses to the lesions were 0.603 (IQR: 0.230–1.810) Gy/GBq and 6.70 (IQR: 3.40–49) Gy/GBq dose per cycle in the [^177^Lu]Lu-DOTA.SA.FAPi, and [^177^Lu]Lu-DOTAGA.(SA.FAPi)_2_ groups, respectively. The first clinical dosimetry study demonstrated significantly higher tumor absorbed doses with [^177^Lu]Lu-DOTAGA.(SA.FAPi)_2_ compared to [^177^Lu]Lu-DOTA.SA.FAPi. [^177^Lu]Lu-DOTAGA.(SA.FAPi)_2_ is safe and unveiled new frontiers to treat various end-stage cancer patients with a theranostic approach.

## 1. Introduction

The tumor microenvironment (TME) plays a crucial role in tumor remodelling and is an important contributor to tumor growth and promoting drug resistance. Within the TME, cancer-associated fibroblasts (CAFs) have a multifaceted function and are major contributors to TME remodelling. The high abundance of CAFs in a wide range of tumors offers important implications to target various cancers.

The fibroblast activation protein (FAPα), a type II transmembrane serine protease, is highly expressed in CAFs. Histopathologic studies reported the prevalence of FAP-positive cancer-associated fibroblasts in ~90% of epithelial tumors [1]. The ubiquitous expression of fibroblast activation protein (FAP) makes it an interesting target for imaging and therapy of a wide spectrum of malignancies [1]. FAP promotes tumor growth, proliferation, and angiogenesis [2]. Hence, targeting this protein with several probes, including antibodies, immunoconjugates, and small molecular FAP inhibitors, may be an interesting approach for tumor detection and suppression. 

**Figure 1:**
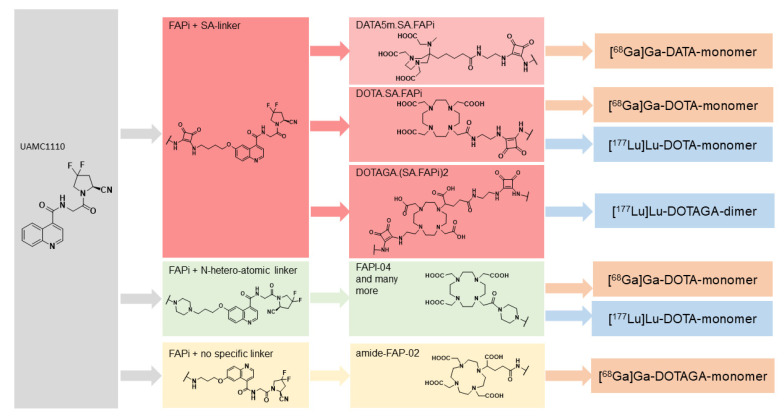
Generations of chelator–linker FAP inhibitor conjugates, a step towards development of FAP-targeted theranostics.

Although FAP imaging is in the early developmental stage, several FAP inhibitor based small molecules (chelator–linker–FAP inhibitor conjugates) have been developed. Mostly, heterocyclic linker units between chelator and inhibitor were introduced, such as piperazine series [3,4] and squaramide based [5,6,7] FAP-inhibitor precursors. All these FAPi precursors exhibit high selectivity and affinities for FAP in the same order of magnitude as the lead structure UAMC1110, as described by van der Veken’s group [8,9], and have been developed for diagnostic and therapeutic use (Figure 1).

Haberkorn’s group reported a series of piperazine-based FAP-inhibitors labelled with the positron emitter gallium-68, which were successfully used for imaging various cancers [3,4], particularly when utilizing the most prominent molecules among their structures such as FAPI-04, FAPI-21, and FAPI-46 [4]. 

Roesch’s group, in collaboration with our group, introduced a modified ligand, keeping the pharmacophore intact as new FAPi PET tracers. The critical subunits constituted the squaramide (SA) linker unit coupled with the DOTA/DATA^5m^ bifunctional chelators and a FAP inhibitor targeting moiety. Both agents were coupled with generator produced gallium-68 and revealed promising imaging and theranostic benefits on in vitro, preclinical, and clinical studies [5,6]. It is well established now that these FAP inhibitors show expression in various cancers [5,6,7,10]. The monomeric DOTA.SA.FAPi labelled with gallium-68 showed the most favourable properties from the imaging point of view, which includes high tumor-to-background ratios [TBR] and demonstrates a great applicability for the theranostic treatment approach for various cancers. 

Our group [7] applied a theranostic approach of [^68^Ga]Ga guided [^177^Lu]Lu-DOTA.SA.FAPi therapy in an advanced stage breast cancer (histology status: ER^−^, PR^−^, HER2/neu^+^) patient who failed multiple lines of treatment, and demonstrated a promising improvement in the quality of life. This radio–ligand therapy concept unveiled a new milestone in precision oncology. However, the findings were preliminary, and the detailed pharmacokinetics and dosimetry data were underway. Visual analysis on PTx-[^177^Lu]Lu-DOTA.SA.SA.FAPi whole-body scan demonstrated a high tumor affinity, but early washout of the radiotracer, which was completely eliminated by 48 h p.i., was the major drawback of the molecule. However, despite the short-tumor retention time, the patient experienced an improvement in the clinical status. 

To overcome this problem, Moon et al. [11] modified the structure and introduced dimeric systems for prolonged tumor retention. Using the SA.FAPi monomer as the base, they developed two homodimeric structures such as DOTA(SA.FAPi)_2_ and DOTAGA.(SA.FAPi)_2_ (Figure 1). 

The DOTAGA.(SA.FAPi)_2_ is based on the monomeric DOTA.SA.FAPi structure, but unlike the monomer, two identical SA.FAPi units are bound to a trifunctional DOTAGA chelator forming a homodimeric system. Additionally, for the possibility of complexing radiometals such as lutetium-177 or actinium-225, at least seven coordinations are required; hence, DOTAGA as a chelator was used in the case of the dimer (Figure 1). 

Various derivatives were investigated in in vitro binding assays to FAP, DPPs (proline-specific enzymes dipeptidyl peptidases), and PREP (prolyl oligopeptidase), and revealed high affinity and protease selectivity to FAP and towards DPPs and PREP (Table 1).

It is of interest to know whether in vitro results of homodimers still hold true from the clinical aspect; therefore, the aim of the present study was to compare the in vivo biodistribution, pharmacokinetics, absorbed dose estimates, and effective half-lives of [^177^Lu]Lu-DOTA.SA.FAPi monomer and [^177^Lu]Lu-DOTAGA.(SA.FAPi)_2_ dimer in cancer patients. 

## 2. Results

### 2.1. Patients 

[^177^Lu]Lu-DOTA.SA.FAPi and [^177^Lu]Lu-DOTAGA.(SA.FAPi)_2_ were administered as a therapy after pretherapeutic confirmation of adequate FAP expression (SUVmax > 3) of the metastases on [^68^Ga]Ga-DOTA.SA.FAPi-PET/CT. The mean SUVmax values and tumor-to-background (pancreas) ratios in both groups were 8.1 ± 0.8 (6.7–9), 4 ± 0.5 (3.3–4.8), and 10.2 ± 2.1 (7.2–14.2), 4.3 ± 1.1 (2.7–6.7), respectively (Tables S1 and S2). 

The demographics of patients treated with [^177^Lu]Lu-DOTA.SA.FAPi and [^177^Lu]Lu-DOTAGA.(SA.FAPi)_2_ are mentioned in Table 2. 

A complete concordance was observed in the FAPi expression of lesions between pre-therapy Dx-[^68^Ga]Ga-DOTA.SA.FAPi PET/CT and post-therapeutic [^177^Lu]Lu-DOTA.SA.FAPi/[^177^Lu]-DOTAGA.(SA.FAPi)_2_ scans (Supplementary Tables S1 and S2). 

Three breast cancer patients from the [^177^Lu]Lu-DOTA.SA.FAPi group (mean age ± SD: 50 ± 17.2 years; range: 31–63 years) were injected with a median cumulative activity of 2.96 GBq (IQR: 2.2–3 GBq). In the [^177^Lu]Lu-DOTAGA.(SA.FAPi)_2_ group, seven patients (mean age ± SD: 51 ± 12.7 years; range: 26–63 years) were injected with a median dosage of 1.48 GBq (IQR: 0.6–1.5 GBq) at the first cycle of treatment. While patients in [^177^Lu]Lu-DOTA.SA.FAPi group received only one treatment cycle, patients in the [^177^Lu]Lu-DOTAGA.(SA.FAPi)_2_ dimer group received two cycles of treatment at a median interval of two months. 

### 2.2. Safety

[^177^Lu]Lu-DOTA.SA.FAPi and [^177^Lu]Lu-DOTAGA.(SA.FAPi)_2_ doses were well tolerated. None of the patients experienced any early adverse events after the administration of the agents. One patient with extensive skeletal metastases and pre-existing grade I anaemia experienced grade III and grade I anaemia and thrombocytopenia, respectively. No other grade III/IV toxicities were noted. Table 3 summarises the median pre-treatment and six months post-treatment hematological, kidney, and liver function parameters that showed that both radiotracers were well endured. 

### 2.3. Biodistribution and Pharmacokinetics of Normal Organs

#### 2.3.1. [^177^Lu]Lu-DOTA.SA.FAPi

**Figure 2:**
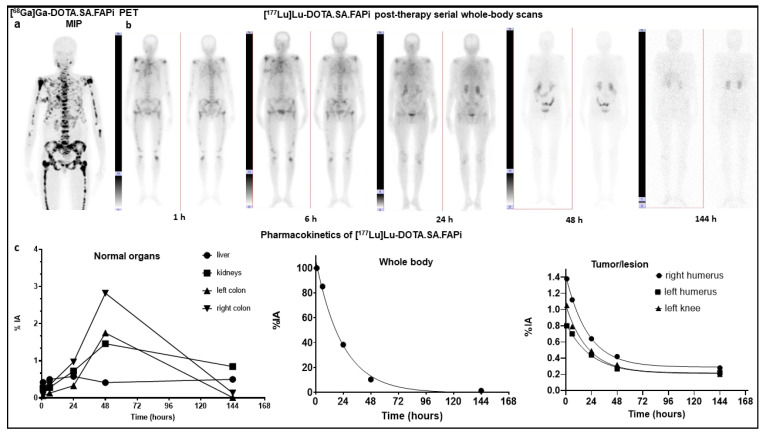
(**a**). [^68^Ga]Ga-DOTA.SA.FAPi PET/CT images of a 49-year-old woman with breast cancer shows biodistribution in the oral mucosa, pancreas, and kidneys and intense expression of DOTA.SA.FAPi in extensive skeletal metastases. B Serial [^177^Lu]Lu-DOTA.SA.FAPi whole body scintigraphy images for dosimetry, after intravenous injection of 1.85 GBq of radiotracer, demonstrates normal and minimal biodistribution in the oral mucosa, salivary glands, liver, kidneys, and intestines. (**b**). Accumulation in the metastatic sites were observed at 1 and 6 h p.i. and decreased significantly by 24 h p.i., with nearly complete washout by 48 h p.i. (**c**). Time–activity curves for whole body and organs that were easily discernible and metastatic sites generated from region of interest placed on whole-body scintigraphy images.

On qualitative analysis, maximum normal physiological uptake was observed in the kidneys, followed by the colon/large intestines (ascending, transverse, and descending colon). Other organs included the liver, pancreas, gall bladder, oral mucosa, lacrimal gland, and urinary bladder contents. Time-activity curves were derived by either mono or biexponential curve fitting. [^177^Lu]Lu-DOTA.SA.FAPi is excreted via both renal excretion and hepatobiliary clearance. A combined wash-in and washout trend of the radiotracer in the kidneys was observed in all patients. The wash-in of the radiotracer in the kidneys was initiated as early as 6 h p.i. and continued up to 144 h after treatment. Pure washout of the trend was observed for lacrimal glands, oral mucosa, liver, and pancreas (Figure 2).

Transit of radiotracer in the gut (ascending and descending colon) was first observed at 24 h post-injection and reached its peak uptake at 48 h. At 144 h p.i., a complete washout of the radiotracer was observed from the gut. Figure 2 shows the [^177^Lu]Lu-DOTA.SA.FAPi serial whole-body scintigraphy images, obtained up to 144 h, of breast cancer patients with extensive skeletal metastases. The image demonstrates the biodistribution of FAPi in various organs. The graphical representation details the pattern of clearance of [^177^Lu]Lu-DOTA.SA.FAPi from the organs and the whole body, which was predominantly bi-phasic (Figure 2).

#### 2.3.2. [^177^Lu]Lu-DOTAGA.(SA.FAPi)_2_

**Figure 3:**
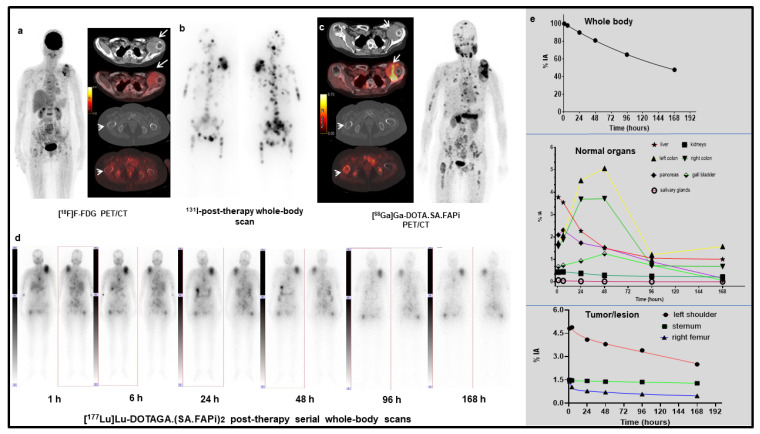
(**a**). [^18^F]F-Fluorodeoxyglucose (FDG) PET/CT images of a 50-year-old woman with follicular variant of papillary carcinoma, post radioiodine therapy (cumulative dose of 22.2 GBq) showing soft tissue density mass in left shoulder (arrows) and multiple skeletal lesions. (**b**). Whole body scintigraphy conducted after additional 7.4 GBq of radioiodine therapy, showing multiple foci of tracer accumulation suggestive of disease progression, who was started on Sorafenib (400 mg OD). (**c**). [^68^Ga]Ga- DOTA.SA.FAPi PET/CT images (performed after 6 months of Sorafenib therapy as part of ongoing clinical study when the patient had clinically progressive disease with thyroglobulin 300,000 ng/mL) show normal biodistribution in the oral mucosa, salivary glands, liver, pancreas, gall bladder, colon, and kidneys. Intense accumulation of radiotracer in the soft tissue mass (arrows) and multiple skeletal sites (right femur-arrow head). (**d**). Serial [^177^Lu]Lu-DOTAGA.(SA.FAPi)_2_ whole-body scintigraphy images for dosimetry, after intravenous injection of 1.48 GBq of radiotracer, showing radiotracer retention in the metastatic sites until 168 h delayed images. (**e**). Time–activity curves for whole body, organs that were easily discernible, and metastatic sites generated from region of interest placed on whole-body scintigraphy images. Accumulation in the normal organs peaked during 24–48 h and decreased significantly by 96 h post injection. Left shoulder, sternum, and right femur show persistent retention until 168 h delayed images. Patient received two cycles of [^177^Lu]Lu-DOTAGA.(SA.FAPi)_2_ therapy and showed significant clinical improvement with a decrease in thyroglobulin levels to 27,000 ng/mL. The patient also showed significant decrease in the VASmax score, from 10 to 5, in a follow-up at 4.5 months.

Physiological biodistribution of [^177^Lu]Lu-DOTAGA.(SA.FAPi)_2_ involved liver, gall bladder, large intestines (transverse, ascending, and descending colon), pancreas, kidneys, urinary bladder contents, and, to a lesser extent, the lacrimal glands, oral mucosa, salivary glands (Figure 3). Visual analysis revealed the colon as the organ with the highest FAP uptake. The route of excretion was predominantly via biliary followed by renal excretion. A pure washout trend was observed by the kidneys, and a combined wash-in and washout trend of radiotracer was observed by the biliary route and fitted with bi-exponential curves. Kidney excretion was seen as early as 1 h p.i. and continued up to 168 h (Figure 3). 

Excretion of radiotracer from the hepatobiliary system/liver commenced at 4 h p.i. and rapidly reduced by 50% at 24 h p.i. (5.2%—1 h p.i. to 2.4%—24 h p.i.); it further reached negligible concentration at 168 h p.i. (1% IA). 

The radiotracer concentration in the colon was observed at 24 h p.i. (mean %IA: 14%), showed the maximum uptake at 48 h p.i. (%IA: 17.8%), and washed out to as low as 3% at 168 h post-infusion. Clearance of gut activity by approximately 7.4-fold was observed between 96 h and 168 h. However, the radiotracer concentration in the gut widely varied among the patients depending on the tumor burden, intestinal motility, and excretion. Patients with a high tumor burden received lower absorbed doses to the colon and kidneys due to the “tumor-sink” effect. 

### 2.4. Dosimetry Estimate and T_e_ of Normal Organs

The mean absorbed doses of [^177^Lu]Lu-DOTA.SA.FAPi and [^177^Lu]Lu-DOTAGA.(SA.FAPi)_2_ are mentioned in Table 4. In the [^177^Lu]Lu-DOTA.SA.FAPi group, the non-target organs with the highest absorbed dose were noted in the kidneys (0.618 ± 0.015 Gy/GBq), followed by a colon (right colon: 0.472 Gy/GBq and left colon: 0.430 Gy/GBq). On the other hand, with [^177^Lu]Lu-DOTAGA.(SA.FAPi)_2_ a colon received the highest absorbed dose (right colon: 1.160 Gy/GBq and left colon: 2.870 Gy/GBq) and demonstrated a significantly higher mean absorbed dose than [^177^Lu]Lu-DOTA.SA.FAPi (*p* < 0.011). The marrow dose was 0.000984 ± 0.000258 and 0.0173 ± 0.0182 Gy/GBq for the [^177^Lu]Lu-DOTA.SA.FAPi, and [^177^Lu]Lu-DOTAGA.(SA.FAPi)_2_ groups, respectively. 

Contrary to [^177^Lu]Lu-DOTA.SA.FAPi, which showed mono-exponential whole-body clearance, [^177^Lu]Lu-DOTAGA.(SA.FAPi)_2_ radiotracer followed a bi-exponential clearance. [^177^Lu]Lu-DOTAGA.(SA.FAPi)_2_ had significantly longer median whole-body T_e_ compared to that of [^177^Lu]Lu-DOTA.SA.FAPi [46.2 h (IQR: 38.5–70.1) vs. 23.1 h (IQR: 17.8–31.5); *p*-0.0167].

### 2.5. Tumor Pharmacokinetics, Effective Half-Lives, and Absorbed Dose Estimate 

The values for the corresponding absorbed doses for various tumor lesions for [^177^Lu]Lu-DOTA.SA.FAPi and [^177^Lu]Lu-DOTAGA.(SA.FAPi)_2_ are presented in Table 5 and Table 6. The median masses of the lesions in the [^177^Lu]Lu-DOTA.SA.FAPi and [^177^Lu]Lu-DOTAGA.(SA.FAPi)_2_ groups were similar: 32 g (IQR:16.8–43.3) and 31.7 g (5.8–111.7), *p*-0.8658, respectively. 

All lesions that showed expression on [^68^Ga]Ga-DOTA.SA.FAPi PET/CT scans were visualized on both [^177^Lu]Lu-DOTA.SA.FAPi and [^177^Lu]Lu-DOTAGA.(SA.FAPi)_2_ post-therapy scans. Uptake in the tumor and metastases was detectable as early as 1 h p.i. for both radiotracers. Interestingly, on qualitative analysis, despite the early and avid uptake of [^177^Lu]Lu-DOTA.SA.FAPi in the tumor lesions, a rapid washout was observed, with only minimal uptake in the lesion at 24 h and no uptake at 48 h post-treatment. In contrast, there were combined wash-in and washout trends observed in the lesions of [^177^Lu]Lu-DOTAGA.(SA.FAPi)_2_ group, where lesions demonstrated avid uptake even up to 168 h post-treatment. Similar to the clearance pattern from the whole body, a rapid mono-exponential clearance was observed with [^177^Lu]Lu-DOTA.SA.FAPi radiotracer compared to the significantly slow and bi-phasic clearance of [^177^Lu]Lu-DOTAGA.(SA.FAPi)_2_ radiotracer. 

In total, all lesions received a median absorbed dose of 0.603 (IQR: 0.230–1.810) Gy/GBq in the [^177^Lu]Lu-DOTA.SA.FAPi group, and patients belonging to the [^177^Lu]Lu-DOTAGA.(SA.FAPi)_2_ group received a significantly higher absorbed dose of 6.70 (IQR: 3.40–49) Gy/GBq dose per cycle (Table 5 and Table 6).

The Te of tumors in both groups reflected the uptake pattern. Unlike [^177^Lu]Lu-DOTA.SA.FAPi, a remarkably higher tumor Te was observed in the patient group treated with [^177^Lu]Lu-DOTAGA.(SA.FAPi)_2_. ^177^Lu]Lu-DOTAGA.(SA.FAPi)_2_: 86.6 h (IQR: 34.3–94.6) vs. [^177^Lu]Lu-DOTA.SA.FAPi: 14 h (IQR: 12.8–15.5); *p*-0.0004 (Table 7).

The absorbed dose to the bone lesions of [^177^Lu]Lu-DOTAGA.(SA.FAPi)_2_ group was about 5.6-fold higher than that in the [^177^Lu]Lu-DOTA.SA.FAPi group, 6.075 (2.520–11.515) Gy/GBq with eight lesions vs. 1.086 (0.540–1.890) Gy/GBq with six lesions, *p*-0.0019). Due to the difference in the type of cancer and the low sample size, the comparison was not possible for other categories of lesions such as primary tumor, lymph nodes, and visceral metastases.

### 2.6. Response Assessment

Patients in the [^177^Lu]Lu-DOTA.SA.FAPi group were administered only a single cycle of treatment, hence one hematological and clinical response was assessed. Though patients showed initial response, which remained for up to six weeks post treatment, they demonstrated relapse in the clinical symptoms. Two patients in this group died.

On the contrary, all patients in the [^177^Lu]Lu-DOTAGA.(SA.FAPi)_2_ group have demonstrated a clinical response, have completed a median of three cycles of treatment, and are alive.

## 3. Discussion

The expression of cancer-associated FAP in a broad spectrum of cancers offers an optimal target for various molecular-based FAP inhibitor imaging and therapies [6,7,12].

Based on the synthesis of a potent FAP inhibitor UAMC1110 [8,9], Moon et al. [5] introduced a squaramide linker containing bifunctional DATA^5m^ and DOTA chelators and a FAP targeting moiety abbreviated as DATA^5m^.SA.FAPi and DOTA.SA.FAPi, that were labelled with gallium-68. Both show sufficient in vitro affinity in nanomolar IC_50_ values for FAP and low affinity in µM IC_50_ ranges for DPPs and PREP. Selectivity to FAP and, accordingly, towards DPPs and PREP is an important aspect for efficacy targeting FAP.

Research on [^68^Ga]Ga-DOTA.SA.FAPi in an HT-29 human colorectal cancer xenograft mouse model and extended clinical studies revealed excellent in vivo and ex vivo results [5].

Subsequently, we conducted clinical studies comparing [^68^Ga]Ga-DOTA.SA.FAPi with [^18^F]F-FDG in various cancers and demonstrated comparable results and complimentary benefits to [^18^F]F-FDG PET/CT reporting, also demonstrating a scope for [^68^Ga]Ga-DOTA.SA.FAPi guided theranostic approach for the treatment of various cancers [6].

Among the various FAP targeted molecules, Haberkorn’s group [3] evaluated FAPI-04 as a theranostic tool. Preclinical studies in cells expressing human and murine FAP and CD26 resulted in an increased half-life of 3.0 h for [^177^Lu]Lu-FAPi-04, versus 1.7 h for [^177^Lu]Lu-FAPi-02 in the tumor. Further, they attempted a theranostic approach of [^68^Ga]Ga-FAPI-04 guided [^90^Y]Y-FAPI-04 therapy in an end-stage breast cancer patient. Though the tumor absorbed dose for [^90^Y]Y-FAPI-04 was not conclusive, the patient experienced a reduction in pain with no significant toxicities [3].

From the reports of previous studies and our study on [^177^Lu]Lu-DOTA.SA.FAPi [3,7], it is evident that the main challenge for the potential therapeutic application of the FAP tracers was to optimize its tumor retention time. To design an ideal radiotracer for theranostic use and deliver a maximum radiation dose to the desired target lesions, the biological half-life of the FAPI agent should match the physical half-life of the radiometal. Small-molecule inhibitors with a shorter-biological half-life could be labelled with shorter physical half-life therapeutic radionuclides such as ^90^Y/^188^Re/^213^Bi, and similar molecules with longer half-life could be tagged to long-lived therapeutic radionuclides such as ^177^Lu/^225^Ac, etc.

This problem received substantial interest, and an approach to improve tumor affinity as well as tumor retention led to the evolution from monomers to dimeric systems, such as DOTA based homodimeric structures DOTA.(SA.FAPi)_2_ and DOTAGA.(SA.FAPi)_2_, by Moon et al. [11]. Unlike monomeric precursors, the bifunctional chelator at the centre is linked to two squaramide linker/target vector units. The coupling of squaramide linker–target vector (FAP inhibitor) to dimers significantly increases tumor uptake, tumor retention, and low background. DOTA.(SA.FAPi)_2_ and DOTAGA.(SA.FAPi)_2_ were synthesized, tested for stability in vitro, and complexed with gallium-68 and lutetium-177. As evidenced from the reports of Moon et al. [11], the homodimers display very high in vitro affinity for FAP, similar to the monomeric structures (similar low nM IC_50_ values). The use of the DOTAGA chelator for the dimeric version allows for the same coordination sites as the DOTA monomer structure, attributing it to the better chelation with heavy radiometals such as ^177^Lu, ^90^Y, ^188^Re, ^225^Ac, ^213^Bi, etc. It increases the tumor retention time by several folds. Interestingly, the homodimeric structure had significantly increased tumor uptake and retention, with a low background at 24 h p.i. compared to the monomer. To introduce the homodimers from bench to bedside, the systematic clinical trials focusing on head-to-head comparisons of the homodimers addressing the pharmacokinetics in normal organ and tumor lesions are warranted.

Both radiotracers were well tolerated in all patients with minimal toxicities. While the dose-limiting organ with [^177^Lu]Lu-DOTA.SA.FAPi was the kidney, followed by a colon, the highest estimated absorbed radiation dose by [^177^Lu]Lu-DOTAGA.(SA.FAPi)_2_ dimer was observed in the colon, followed by gall bladder, pancreas, and kidneys.

To achieve a safe limit of 28 Gy [13] to the kidneys, a calculated maximum cumulative activity of 45 GBq [^177^Lu]Lu-DOTA.SA.FAPi and ~10 GBq [^177^Lu]Lu-DOTAGA.(SA.FAPi)_2_ can be safely administered. A study by Bodei et al. [14] has followed patients post-PRRT in NET patients and revealed that long-term kidney toxicities are minimal. The safe, tolerable limit of [^177^Lu]Lu-DOTATATE or [^90^Y]Y-DOTATOC could reach up to 40 Gy in patients who have no prior risk factors, co-morbidities, or previous history of impaired kidney function.

**Figure 4:**
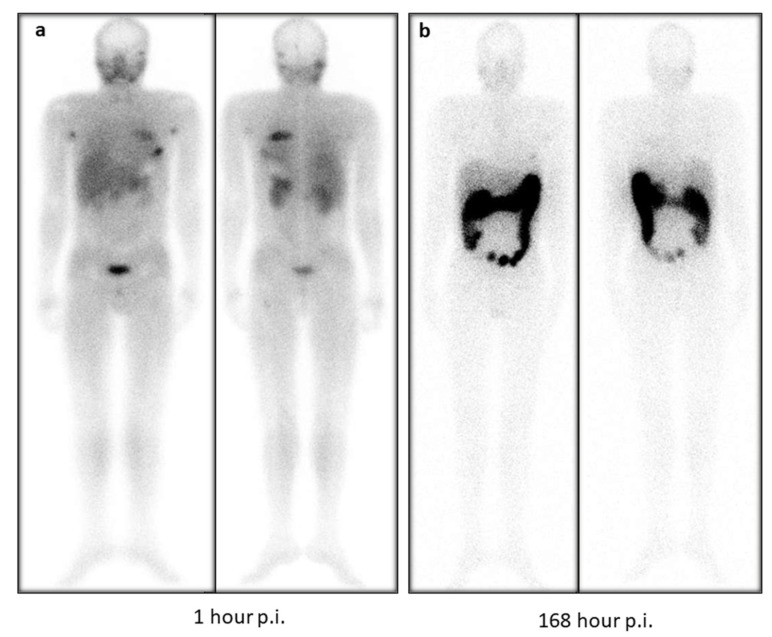
(**a**). A 27-year old male diagnosed with paraganglioma was treated with [^177^Lu]Lu-DOTAGA.(SA.FAPi)_2_ treatment, and the 1 h scan post-treatment showed (**a**) normal and minimal biodistribution of radiotracer in the oral mucosa, salivary glands, liver, pancreas, and kidneys. At 1 h p.i., intense accumulation of radiotracer was observed in the skull and rib lesions. The patient has a history of constipation and hence demonstrated persistent and intense uptake of [^177^Lu]Lu-DOTAGA.(SA.FAPi)_2_ radiotracer in the gut due to reduced intestinal motility, even at 168 h (**b**) post-treatment, reflecting higher radiation absorbed dose to the colon compared to the other patients, such the patient treated in Figure 3.

Based on the maximum tolerable dose limit of 38 Gy in the colon, based on the stereotactic body radiation therapy (SBRT) data [15], patients can be injected with as much as 84 GBq of [^177^Lu]Lu-DOTA.SA.FAPi and approximately 20 GBq of [^177^Lu]Lu-DOTAGA.(SA.FAPi)_2_. Among our patient series in the [^177^Lu]Lu-DOTAGA.(SA.FAPi)_2_ group, one patient with paraganglioma suffered from constipation, with the persistence of activity in the gut even up to 168 h p.i., and hence received a relatively higher dose absorbed to the colon (Figure 4). The physiological uptake in the gut/intestines varied widely across the patients and was majorly dependent on intestinal motility. Efforts to reduce the risk to the colon and reduce the absorption might be facilitated by suggesting that high fatty food to accelerate the washout from the gall bladder and administration of laxatives to accelerate the washout of the radiotracer from the gut may be beneficial. However, the effect of the above methods cannot be deduced from the current study results, and it mandates a proper execution and investigation.

The median absorbed dose to the tumor lesions was 6.70 Gy/GBq in patients injected with [^177^Lu]Lu-DOTAGA.(SA.FAPi)_2,_ which was 5.16-fold higher than that deposited by [^177^Lu]Lu-DOTA.SA.FAPi (0.603 Gy/GBq). Comparable tumor absorbed doses of 6.64 Gy/GBq were obtained by Kramer et al. in the case of [^177^Lu]Lu-PSMA-ALB-56 [16]. The high absorbed doses from [^177^Lu]Lu-DOTAGA.(SA.FAPi)_2_ were in concordance with the high survival rate of advanced stage disease in our patient cohort.

[^177^Lu]Lu-DOTAGA.(SA.FAPi)_2_ demonstrates rapid internalization, high tumor uptake, prolonged tumor effective half-life, and delivers high radiation dose to the tumors, even with lower dosages of lutetium. However, along with providing promisingly high tumor doses, [^177^Lu]Lu-DOTAGA.(SA.FAPi)_2_ also attributes higher absorbed dose to the whole body, including other organs at risk such as the gall bladder, pancreas, kidneys, and liver.

It should be underlined that the benefit-to-risk ratio should be weighed for each patient, taking into account factors such as tumor burden, the bowel emptying time of each patient, and history of hepatobiliary obstruction. Additionally, dose fractionation protocols by inducing a small dose of [^177^Lu]Lu-DOTAGA.(SA.FAPi)_2_ per treatment cycle at longer treatment intervals may cover a decent treatment period, and at the same time induce tumor regression and provide a window for recovery of vital and clinical toxicities.

### 3.1. Limitations

The stutandardizses certain limitations. The number of patients in the study is small and varied between the groups (three vs. seven). As FAPi based radionuclide therapy is a new treatment, the institute ethics committee approved the [^177^Lu]Lu-labelled FAPi derivatives as a salvage treatment option. Hence, only the worst prognosis patients who have exhausted all standard line treatment options were recruited and were treated on compassionate grounds, causing a bias in the patient recruitment. The current administered activities to patients were arbitrary, as no data on the dosimetry estimates were available in the literature for any therapeutic FAPi tracer.

Pertaining to the heterogeneity in the type of cancers and difference in the tumor burdens of the patients between the two radiotracer groups, it is not ideal for conducting a head-to-head comparison. The serial time-point of the acquisition was not uniform due to the differences in the pharmacokinetics between the radiotracers. An inherent drawback of planar dosimetry is the overestimation of dose due to the overlap of abdominal organs, but efforts were made to reduce the error by applying appropriate subtraction techniques. The effect of laxatives to promote the early washout of radiotracers and the reduction in radiation burden to the large intestine was out of the scope of this paper.

### 3.2. Future Prospects

Based on the current results, we have initiated a dose-escalation study to evaluate the maximum tolerated absorbed dose to critical organs for [^177^Lu]Lu-DOTAGA.(SA.FAPi)_2_; thereby, we expect to achieve the best objective response and minimal toxicities at an optimal dosage of lutetium-177.

From the molecular perspective, we intend to reconstruct/improvise the molecule further, improve its pharmacokinetics to promote minimum uptake in the non-target organs by reducing the percentage of injected activity to the dose-limiting organs (hepatobiliary and large bowels), and enhance tumor internalization and achieve greater “tumor-sink” effect.

## 4. Materials and Methods

### 4.1. Patient Recruitment

The study was duly approved by the ethics committee of the All India Institute of Medical Sciences, New Delhi as a salvage treatment option and for patients who have exhausted standard line treatment option on compassionate grounds. Patients were included for [^177^Lu]Lu-DOTA.SA.FAPi or [^177^Lu]Lu-DOTAGA.(SA.FAPi)_2_ treatment if they had histologically confirmed carcinoma, documented radiological/molecular or biochemical disease progression on previous lines of treatment, and have exhausted all lines of treatments, with ECOG status up to four, cancers that demonstrated high FAPi expression on [^68^Ga]Ga-DOTA.SA.FAPi PET/CT scan (SUVmax > 3), and patients who signed the informed consent form.

Patients who received prior anti-cancer therapy within the previous four weeks, patients with Hb < 9 g/dL, leukocyte counts less than 4.0 × 10^9^/L, platelet counts less than 75,000 per mL, inadequate liver function parameters, and serum creatinine > 1.2 mg/dL were excluded from the study. 

The study was first initiated using [^177^Lu]Lu-DOTA.SA.FAPi, but after the preliminary qualitative results of serial imaging, we observed low radiotracer retention at about one to two days p.i. in the target lesions. To improve the radiotracer’s retention time, further modifications of the radiopharmaceutical’s design led to the development of DOTAGA.(SA.FAPi)_2_ homodimer.

Pertaining to the time difference in chemical modifications in the molecule, the recruiting time-points in both patient groups were different. A total of three patients (mean: 50 ± 17.2 (31–63) years, three females) were recruited from May 2020 to August 2020 in the [^177^Lu]Lu-DOTA.SA.FAPi group. Seven patients (mean: 51 ± 12.7 (26–63) years, four males and three females) were recruited between November 2020 and March 2021 in the [^177^Lu]Lu-DOTAGA.(SA.FAPi)_2_ group. Dosimetry analysis was conducted, compared, and analyzed between patients treated with [^177^Lu]Lu-DOTA.SA.FAPi and [^177^Lu]Lu-DOTAGA.(SA.FAPi)_2_. 

### 4.2. [^68^Ga]Ga-DOTA.SA.FAPi PET/CT Imaging

Scans were obtained on a dedicated GE Discovery 710* 128 Slice PET/CT Scanner (Wipro GE Healthcare Private Limited, 1, Maritime Square, Singapore) with a 40-mm detector at a rotation speed of 0.35 s. Whole-body PET/CT scans were acquired 1 h after the administration of [^68^Ga]Ga-DOTA.SA.FAPi (mean injected activity: 148 MBq). Patients were positioned in a supine position, and an initial scout was acquired, followed by a diagnostic dose CT with 300–350 mAs, 120 kVp, slice thickness 5 mm, and pitch 1 and PET acquisition with 2 min per bed. 

The images were subjected to dead-time, random, and scatter correction. The PET image reconstruction was performed using an ordered subset expectation maximization algorithm (OSEM) (21 subsets, 3 iterations). All images were processed and analyzed on the GE Xeleris workstation.

### 4.3. [^177^Lu]Lu-DOTA.SA.FAPi and [^177^Lu]Lu-DOTAGA.(SA.FAPi)_2_ Radiolabelling

DOTA.SA.FAPi and DOTAGA.(SA.FAPi)_2_ (25 nmol) were radiolabelled with [^177^Lu]LuCl_3_, which was obtained from BRIT, India, in sodium acetate buffer, pH 4, in 0.01 M supra pure HCl. The radiolabelled solution was heated at 95 °C for 30 min, followed by purification through Sep-Pak C18 light cartridge and eluted with 50% ethanol. Radiochemical quality control was carried out using the instant thin-layer chromatography method with sodium citrate buffer as the solvent, and radiolabelled products with 95% to 98% purity were administered. 

### 4.4. Post-Therapy [^177^Lu]Lu-DOTA.SA.FAPi and [^177^Lu]Lu-DOTAGA.(SA.FAPi)_2_ Whole Body Scintigraphy

The planar acquisition of whole-body scans was performed using a dual-headed gamma camera (GE, Discovery NM/CT 670). The camera was equipped with a high-energy general-purpose (HEGP) parallel-hole collimator, and the energy peak was centred at 113 keV and 208 keV with a 10% window width. Dual-energy scatter corrections were applied at 90 KeV and 170 keV with a window width of 10%. Serial whole-body emission scans were performed at 1 (pre-void), 6, 24, 48, and 144 hours (h) after treatment for the [^177^Lu]Lu-DOTA.SA.FAPi group and at 1 (pre-void), 4–6, 24, 48, 96, and 144–168 h in the [^177^Lu]Lu-DOTAGA.(SA.FAPi)_2_ group. Simultaneous anterior and posterior emission scans were acquired at a speed of 15 cm/min and a matrix size of 256 × 1024. Delayed images were acquired up to 168 h post-injection to prevent the overestimation of doses.

Similarly, SPECT/CT scans of the abdomen and the lesions were acquired in both the radiotracer groups at serial time points, but were mainly used to demarcate the overlapping gut and kidney activity and to calculate the volume of the tumor. SPECT/CT acquisition parameters included a total angular range of 360 degrees, an angle view of 6 degrees, acquired at 25 s per view, and a matrix size of 512 × 512. 

### 4.5. Image Analysis

In the dosimetry analysis, salivary glands, kidneys, pancreas, liver, gall bladder, right colon, left colon, tumor lesions, and whole body were included for dose calculation. The first whole-body image post-injection before voiding was considered to include 100% of injected activity. The region of interest (ROI) was drawn on the source organs showing uptake of [^177^Lu]Lu-DOTA.SA.FAPi and [^177^Lu]Lu-DOTAGA.(SA.FAPi)_2_ on both anterior (A) and posterior images (P). The ROI of the initial scan was cloned to the subsequent serial time-point images of the patient. 

Background counts were obtained from the thigh region. For overlapping organs such as the right kidney which had overlapping intestinal uptake, the counts were considered to the left kidney. The corresponding time-point post-therapy single photon emission computed tomography (PTx-SPECT/CT) scans were also referred to prevent overlap. Background correction of lesion counts was performed by subtracting counts in background ROI of the similar area drawn close to the lesions. 

Finally, attenuated, background, and scatter corrected percentage injected activity (%IA) in each source organ including salivary glands (parotid and submandibular glands), kidney, liver, gall bladder, pancreas, right and left colon, and the tumor was calculated was calculated according to the Equations (1) and (2).

(1) $$\%IA_{uncorr}=\frac{Ct_{ROI}/pixel}{Ct_{WB}/pixel}\times 100$$

where %*IA_uncorr_*: Uncorrected percentage of injected activity; *Ct_ROI/pixel_*: counts/pixel in a region of interest; *Ct_WB/pixel_*: counts in the whole-body image.

(2) $$\%IA_{Corr}=\frac{Ct_{ROI}/pixel}{Ct_{WB}/pixel}\times DF\times 100$$

where %*IA_Corr_*: Corrected percentage of injected activity (corrected with decay factor); *Ct_ROI/pixel_*: counts/pixel in the region of interest; *Ct_WB/pixel_*: counts/pixel in whole-body image; *DF*: decay factor.

### 4.6. Internal Dose Estimation

The percentage injected activities against time were entered in the kinetic input model of the OLINDA/EXM v2.2 software to calculate the area under the curve that represented the number of disintegrations per injected activity, residence time, or cumulative activity in each source organ or time-integrated activity coefficient. The residence times were input to the ICRP-89 female and male models to derive absorbed doses of organs and whole-body.

### 4.7. Tumor Dosimetry

For the tumor dosimetry, a sphere model implemented within OLINDA/EXM v2.2 was used. For each considered lesion, the volume was evaluated on pre-therapy [^68^Ga]Ga-DOTA.SA.FAPi PET/CT and PTx SPECT-CT of the area of interest using the commercially available workstation (GE Xeleris).

For the estimation of tumor absorbed dose, the dose equation based on the MIRD formalism is expressed below [17,18] [Equation (3)].

(3) $$D=\tilde{A}\;\times \;S=A_{0}\;\times \;\tau \;\times \;S$$

Here, τ is the residence time, $\tilde{A}$ is the cumulated activity, A_0_ is the patient’s administered activity, and S is the mean absorbed dose per unit cumulated activity.

Finally, the residence times of source organs and tumors were entered in the adult female or male ICRP 89 model for normal organs and the sphere model, respectively, that derived the organ and whole-body absorbed doses in terms of or Gy/GBq. The effective half-lives (T_e_) of various organs and tumors were generated using GraphPad Prism software (v9.1).

### 4.8. Lood Dosimetry

Blood dosimetry was conducted in all patients belonging to the [^177^Lu]Lu-DOTA.SA.FAPi and was feasible only in three patients in the [^177^Lu]Lu-DOTAGA.(SA.FAPi)_2_ group. One milliliter of venous blood sample was taken at 0.5 (prevoid), 3.5, 24, 48, 72, 96, 120, 144, and 168 h after injection from each patient. The marrow dose was derived using the method of Sgouros [19].

### 4.9. Safety

Safety was assessed by dosimetry and adverse events assessment according to the National Cancer Institute’s Common Toxicity Criteria (NCI-CTCAE) version 5.0.

### 4.10. Statistical Analysis

The D’Agostino Pearson test was used to check for the normal distribution of data. Based on the distribution, summary statistics were obtained in terms of mean, median, standard deviation (SD), range, and interquartile range (IQR) were calculated for all continuous variables based on the distribution of data. The Mann–Whitney test for independent samples was used to compare the organ, tumor absorbed doses, and the Te between the radiotracers. *p*-value < 0.05 was considered statistically significant. Statistical analysis was performed with MedCalc statistical software version 12. 

## 5. Conclusions

Compared to the [^177^Lu]Lu-DOTA.SA.FAPi monomer, [^177^Lu]Lu-DOTAGA.(SA.FAPi)_2_ homodimer demonstrated a significantly longer tumor retention; however, later uptakes in colon and kidneys are higher than in the former, but are well tolerated. The desired qualities, such as rapid internalization, higher affinity, longer tumor retention, and faster clearance from the non-target organs with [^177^Lu]Lu-DOTAGA.(SA.FAPi)_2_ unveiled new frontiers for the treatment of various end-stage cancer patients with a theranostic approach.

## Figures and Tables

**Table 1:** IC_50_ values for FAP and the related serine proteases (PREP, DPP4, DPP8, DPP9). (Adapted from ref. [5,11]).

Compound	IC_50_ (µM)				IC_50_ (nM)
	DPP4	DPP8	DPP9	PREP	FAP
DOTAGA.(SA.FAPi)_2_	0.4 ± 0.07	0.42 ± 0.04	0.16 ± 0.02	0.39 ± 0.02	0.92 ± 0.06
[^nat^Lu]Lu-DOTAGA.(SA.FAPi)_2_	0.63 ± 0.07	0.41 ± 0.03	0.18 ± 0.02	0.56 ± 0.04	1.54 ± 0.15
[^nat^Ga]Ga-DOTA.SA.FAPi	>1	N/A	>1	8.7 ± 0.9	1.4 ± 0.2
[^nat^Lu]Lu-DOTA.SA.FAPi	>1	N/A	>1	2.5 ± 0.4	0.8 ± 0.2

FAP Fibroblast Activation Protein; DPPs Proline-specific enzymes Dipeptidyl Peptidases; PREP Prolyl Oligopeptidase.

**Table 2:** Patient demographics.

Parameters	[^177^Lu]Lu-DOTA.SA.FAPi	[^177^Lu]Lu-DOTAGA.(SA.FAPi)_2_
**Number of patients**	3	7
**Age (years) [mean ±SD; range]**	50 ± 17.2 (31–63)	51 ± 12.7 (26–63)
Gender		
Male	0	4
Female	3	3
**Type of cancer**		
Breast cancer	3	1
Thyroid cancer	0	5
Paraganglioma	0	1
**The extent of disease on [^68^Ga]Ga-DOTA.SA.FAPi PET/CT scan**		
Primary	1	0
Lymph nodes	1	3
Skeletal metastases	3	4
Brain metastases	1	0
Liver metastases	1	1
Lung mass	0	1
**Injected activity (GBq; IQR)**	2.96 GBq (IQR: 2.2–3 GBq)	1.48 GBq (IQR: 0.6–1.5)

**Table 3:** Comparison of hematological, renal, and liver function parameters pre- and at median of 6 months post-treatment.

[^177^Lu]Lu-DOTA.SA.FAPi Group				[^177^Lu]Lu-DOTAGA.(SA.FAPi)_2_ Group		
Parameters	Baseline (Mean, 95% CI of Mean)	Post-Treatment (Mean, 95% CI of Mean)	*p* Value	Baseline (Mean, 95% CI of Mean)	Post-Treatment (Mean, 95% CI of Mean)	*p* Value
Haemoglobin (g/dL)	10.9 (8.9–11.7)	11 (8.7–11.6)	-	10.8 (9.1–11.9)	10.07 (6.9–11)	0.1121
Platelets (lakhs/µL)	199 (178–201)	199 (188–201)	-	225 (156–295)	198 (81–239)	0.2554
Leukocytes 109/L	6500 (5600–7800)	7700 (6780–7877)	-	6781.6 (4216–9348)	6947.4 (5239–8655)	0.8571
Creatinine (mg/dL)	0.8 (0.7–0.9)	0.77 (0.7–0.9)	-	0.70 (0.2–1.2)	0.50 (0.3–0.64)	0.3853
ALP	212 (168–225)	188 (160–234)	-	98.3 (73.4–123)	90.5 (70.6–110)	0.3042

(-): *p* value cannot be estimated due to low sample size; CI: confidence interval.

**Table 4:** Absorbed dose estimates of [^177^Lu]Lu-DOTA.SA.FAPi and [^177^Lu]Lu-DOTAGA.(SA.FAPi)_2._

[^177^Lu]Lu-DOTA.SA.FAPi		[^177^Lu]Lu-DOTAGA.(SA.FAPi)_2_	
Organ	Mean Absorbed Doses (Gy/GBq)	Organ	Mean Absorbed Doses (Gy/GBq)
**Adrenals**	7.79E-03 ± 3.69E-04	**Adrenals**	1.27E-02 ± 4.63E-03
**Brain**	2.06E-05 ± 2.09E-05	**Brain**	1.17E-04 ± 3.93E-05
**Breasts**	4.39E-04 ± 2.64E-04	**Breasts**	6.39E-04 ± 5.30E-05
**Esophagus**	1.80E-03 ± 9.12E-04	**Esophagus**	3.07E-03 ± 6.35E-04
**Eyes**	2.13E-05 ± 2.04E-05	**Eyes**	9.96E-05 ± 4.11E-05
**Gallbladder Wall**	6.06E-03 ± 1.91E-04	**Gallbladder Wall**	7.95E-01 ± 2.58E-01
**Left colon**	4.30E-01 ± 9.40E-02	**Left colon**	2.87E+00 ± 1.74E+00
**Small Intestine**	2.71E-03 ± 1.74E-04	**Small Intestine**	9.24E-03 ± 4.92E-03
**Stomach Wall**	2.03E-03 ± 3.74E-04	**Stomach Wall**	6.62E-03 ± 1.84E-03
**Right Colon**	4.72E-01 ± 3.93E-02	**Right Colon**	1.16E+00 ± 8.58E-01
**Rectum**	5.08E-04 ± 5.31E-05	**Rectum**	2.02E-03 ± 1.04E-03
**Heart Wall**	1.40E-03 ± 1.09E-03	**Heart Wall**	2.57E-03 ± 1.39E-03
**Kidneys**	6.18E-01 ± 1.54E-02	**Kidneys**	3.74E-01 ± 2.57E-01
**Liver**	1.15E-01 ± 9.02E-03	**Liver**	2.09E-01 ± 2.38E-02
**Lungs**	6.10E-02 ± 1.04E-01	**Lungs**	2.21E-03 ± 5.66E-04
**Ovaries**	9.08E-04 ± 7.3E-05	**Ovaries**	2.23E-03 ± 2.50E-04
**Pancreas**	3.69E-03 ± 2.33E-04	**Pancreas**	6.51E-01 ± 1.37E-01
**Prostate**	-	**Prostate**	2.57E-03 ± 1.35E-03
**Salivary glands**	6.56E-05 ± 6.79E-05	**Salivary glands**	1.17E-01 ± 9.53E-03
**Red Marrow**	9.84E-04 ± 2.58E-04	**Red Marrow**	1.73E-02 ± 1.82E-02
**Osteogenic Cells**	1.18E-03 ± 2.89E-04	**Osteogenic Cells**	8.57E-03 ± 6.95E-03
**Spleen**	3.99E-03 ± 2.18E-04	**Spleen**	6.36E-03 ± 1.52E-03
**Testes**	-	**Testes**	1.71E-04 ± 9.97E-05
**Thymus**	1.22E-03 ± 1.49E-03	**Thymus**	1.00E-03 ± 3.09E-04
**Thyroid**	4.53E-04 ± 5.25E-04	**Thyroid**	3.98E-04 ± 8.53E-05
**Urinary Bladder Wall**	4.05E-04 ± 4.12E-05	**Urinary Bladder Wall**	1.28E-03 ± 4.29E-04
**Uterus**	7.84E-04 ± 7.47E-04	**Uterus**	2.18E-03 ± 2.70E-04
**Total Body**	1.10E-02 ± 1.72E-03	**Total Body**	2.33E-02 ± 6.15E-03

All values are mentioned as mean ± SD.

**Table 5:** Effective half-life (T_e_) and dosimetry estimate of tumor lesions with [^177^Lu]Lu-DOTA.SA.FAPi.

Patient S.No	Cancer Type	Site of Lesion	T_e_ Tumor (h)	No of Disintegrations or Residence Time	Mass of Lesion (g)	Absorbed Dose (Gy/GBq)
1.	Right breast cancer	Right breast primary tumor	17	2.44E+00	800	2.52E-01
		Right shoulder skeletal lesion	13.7	3.09E-01	46.1	5.40E-01
2.	B/L breast cancer	Right shoulder skeletal lesion	14	4.30E-01	22	1.57E+00
		Left shoulder skeletal lesion	16	3.54E-01	15	1.89E+00
		Left knee skeletal lesion	14	0.41E-01	15	2.13E+00
3.	Right breast cancer	Ileum	12.6	2.80E-01	32	6.03E-01
		Pubis	12	1.02E-01	35	2.34E-01
**Total number of lesions**		**7**				
**Median (IQR)**			**14 ** **(12.8–15.5)**	**3.00E-01** **(1.46E-01–4.11E-01)**	**32** **(16.8–43.3)**	**6.03E-01** **(2.30E-01–1.81E+00)**

**Table 6:** Effective half-life (T_e_) and dosimetry estimate of tumor lesions with [^177^Lu]Lu-DOTAGA.(SA.FAPi)_2_.

Patient S.No	Cancer Type	Site of Lesion	Te in Tumor (h)	Number of Disintegrations or Residence Time	Mass of Lesion (g)	Absorbed Dose (Gy/GBq)
1	Radioiodine refractory follicular thyroid cancer	Right ileum skeletal lesion	99	3.37E+00	65.4	4.16E+00
		Femur bone lesion	231	9.80E+00	158	7.99E+00
2	Triple negative breast cancer	Right lung mass	40.7	3.47E+00	50.7	5.51E+00
3	Radioiodine refractory papillary thyroid cancer	Right lung nodule	86.6	6.02E+00	1.5	3.17E+02
4	Radioiodine refractory papillary thyroid cancer	Left shoulder bone lesion	86.6	6.41E+00	189	2.64E+00
		Sternum	89.5	4.47E+00	3.96	8.97E+01
		Right head of femur lesion	48.6	3.97E+00	23.2	1.37E+01
5	Paraganglioma	Skull	27.7	1.82E-01	6	2.40E+00
		Anterior rib lesion	27.9	6.48E-01	5.6	9.33E+00
		Posterior rib lesion	23.9	8.51E-01	30	2.28E+00
6	Anaplastic thyroid cancer	Right Neck mass	90.2	1.54E+01	250	5.02E+00
7	Medullary thyroid cancer	Liver lesion	115.5	3.51E+01	33.4	8.44E+01
**Total number of lesions**		**12**				
**Median (IQR)**			**86.6** **(34.3–94.6)**	**4.22E+00** **(2.11E+0–8.10 E+00)**	**31.7** **(5.8–111.7)**	**6.70E+00** **(3.40E+00 to 4.9E+01)**

**Table 7:** Comparison of T_e_ between [^177^Lu]Lu-DOTA.SA.FAPi and [^177^Lu]Lu-DOTAGA.(SA.FAPi)_2_ post-therapy scans.

T_e_	[^177^Lu]Lu-DOTA.SA.FAPi	[^177^Lu]Lu-DOTAGA.(SA.FAPi)_2_	*p*-Value
**Whole body T_e_**	N = 3 patients	N = 7 patients	
Median (IQR)	23.1 (17.8–31.5)	46.2 (38.5–70.1)	0.0167
**Tumor T_e_**	N = 7 lesions	N = 12 lesions	
Median (IQR)	14 (12.8–15.5)	86.6 (34.3–94.6)	0.0004

## Data Availability

Data is contained within the article and supplementary material.

## Supplementary Materials

The following are available online at https://www.mdpi.com/article/10.3390/ph14121212/s1, Table S1: SUVmax of tumor lesions on the baseline [68Ga]Ga-DOTA.SA.FAPi PET/CT in the [177Lu]Lu-DOTA.SA.FAPi group, Table S2: SUVmax of tumor lesions on the baseline [68Ga]Ga-DOTA.SA.FAPi PET/CT in the [177Lu]Lu-DOTAGA.(SA.FAPi)2 group.
