# Supplementary material for: First-In-Human Results on the Biodistribution, Pharmacokinetics, and Dosimetry of [177Lu]Lu-DOTA.SA.FAPi and [177Lu]Lu-DOTAGA.(SA.FAPi)2
Source: Pharmaceuticals (Basel). 2021 Nov 24;14(12):1212. doi: 10.3390/ph14121212 (PMC8707268; doi:10.3390/ph14121212)
Supplement: Supplementary file 1 [file pharmaceuticals-14-01212-s001.zip › pharmaceuticals-1451036-SI.pdf]

Table S1. SUVmax of tumor lesions on the baseline [^{68}Ga]Ga-DOTA.SA.FAPi PET/CT in the [^{177}Lu]Lu-DOTA.SA.FAPi group.

Patient S.No	Cancer type	Site of lesion	SUVmax	Tumor-to-pancreas ratio
1.	Right breast cancer	Right breast primary tumor	12.5	4.8
		Right shoulder skeletal lesion	8.7	3.3
2.	B/L breast cancer	Right shoulder skeletal lesion	7.8	3.9
		Left shoulder skeletal lesion	8.8	4.4
		Left knee skeletal lesion	6.7	3.4
3.	Right breast cancer	Ileum	8.1	4
		Pubis	9.0	4.5
**Total number of lesions**		**7**		
**Mean±SD (Range)**			**8.1 ± 0.8 (6.7 – 9)**	**4 ± 0.5 (3.3 – 4.8)**

*As pancreas demonstrated highest FAPi expression on [^{68}Ga]Ga-DOTA.SA.FAPi PET/CT tumor-to-pancreas ratio was considered.

Table S2. SUVmax of tumor lesions on the baseline [^{68}Ga]Ga-DOTA.SA.FAPi PET/CT in the[^{177}Lu]Lu-DOTAGA.(SA.FAPi)$_2$ group

Patient S.No	Cancer type	Site of lesion	SUVmax	Tumor-to-pancreas ratio
1	Radioiodine refractory follicular thyroid cancer	Right ileum skeletal lesion	10	3.8
		Femur bone lesion	12.8	6
2	Triple negative breast cancer	Right lung mass	11.1	4.6
3	Radioiodine refractory papillary thyroid cancer	Right lung nodule	14.2	6.7
4	Radioiodine refractory papillary thyroid cancer	Left shoulder bone lesion	9.21	4.6
		Sternum	8.9	4.2
		Right head of femur lesion	7.6	3.8
5	Paraganglioma	Skull	12	4.6
		Anterior rib lesion	8.7	3.3
		Posterior rib lesion	7.2	2.7
6	Anaplastic thyroid cancer	Right Neck mass	9.8	3.6
7	Medullary thyroid cancer	Liver lesion	11.8	4.0
**Total number of lesions**		**N =12**		
**Mean ± SD (Range)**			**10.2 ± 2.1 (7.2 – 14.2)**	**4.3 ± 1.1 (2.7 – 6.7)**
